# Studies to Prevent Degradation of Recombinant Fc-Fusion Protein Expressed in Mammalian Cell Line and Protein Characterization

**DOI:** 10.3390/ijms17060913

**Published:** 2016-06-09

**Authors:** Sanjukta Chakrabarti, Colin J. Barrow, Rupinder K. Kanwar, Venkata Ramana, Jagat R. Kanwar

**Affiliations:** 1 Reliance Life Sciences, Dhirubhai Ambani Life Sciences Center, Navi Mumbai 400701, India; sanjukta_chakrabarti@relbio.com; 2 School of Life and Environmental Sciences, Deakin University, Waurn Ponds Campus, Geelong 3216, Australia; colin.barrow@deakin.edu.au; 3 Nanomedicine-Laboratory of Immunology and Molecular Biomedical Research (NLIMBR), School of Medicine (SoM), Centre for Molecular and Medical Research (C-MMR), Deakin University, Waurn Ponds Campus, Geelong 3216, Australia; rupinder.kanwar@deakin.edu.au

**Keywords:** Fc fusion protein, VEGFR1(D1–D3)-Fc, CHOK1SV GS-KO, clipping, proteolysis, inhibition, stable, folded, biological activity

## Abstract

Clipping of recombinant proteins is a major issue in animal cell cultures. A recombinant Fc-fusion protein, VEGFR1(D1–D3)-Fc expressed in CHOK1SV GS-KO cells was observed to be undergoing clippings in lab scale cultures. Partial cleaving of expressed protein initiated early on in cell culture and was observed to increase over time in culture and also on storage. In this study, a few parameters were explored in a bid to inhibit clipping in the fusion protein The effects of culture temperature, duration of culture, the addition of an anti-clumping agent, ferric citrate and use of protease inhibitor cocktail on inhibition of proteolysis of the Fc fusion were studied. Lowering of culture temperature from 37 to 30 °C alone appears to be the best solution for reducing protein degradation from the quality, cost and regulatory points of view. The obtained Fc protein was characterized and found to be in its stable folded state, exhibiting a high affinity for its ligand and also biological and functional activities.

## 1. Introduction

Fusion proteins are generated by combining the genes encoding two or more proteins, where the benefits of both the components are achieved [1]. Clinically and commercially, the most successful fusion proteins to date are the Fc-fusion proteins. With six fusion proteins in different phases of clinical trials, as of 2014, and several more in the pre-clinical pipeline, it is one of the most expanding and important classes of molecules researchers are focusing on. Most protein drugs have a short serum half-life of a few minutes to a few hours, as a result of large size and rapid renal clearance [2,3]. The Fc region of a Fc-fusion protein confers additional pharmacokinetic (immunoglobulin-like) properties, such as increasing the drug half-life due to interaction with the cellular Fc receptor (FcRn), and serum stability of most biologically active proteins. On the other hand, the fusion partner of the Fc domain imparts the therapeutic benefit of the protein. The sales of approved protein-based drugs in 2010 was approximately USD $108 billion [4], with half of it accounting for monoclonal antibodies and the remaining for Fc fusions, antibody conjugated drugs, bispecific antibodies, antigen-binding antibody fragments, and other engineered therapeutic molecules. In fact, the total revenue generated from therapeutic proteins, including monoclonal antibodies and fusion proteins, in the US is about $64 billion [5]. Global sales for etanercept, the most commercially successful Fc-fusion was USD $7.3 billion in 2010, which was better than that of the most successful IgGs, including bevacizumab ($6.9 billion), rituximab ($6.8 billion) or infliximab ($6.5 billion) [6]. In fact, in 2012, US sales for Fc fusions grew at the highest rate for all bio-therapeutics in the USA, at an incredible ~35.3% (USD $5.8 billion) [7]. There are 47 monoclonal antibodies and Mab-derived products that have been approved in the USA or Europe as of 2014, with 44 being produced in mammalian cells. Of these, 10 belong to the class of Fc fusion proteins. In order to support the ever-increasing need for highly potent bio-therapeutics, the requirement of high drug dosages and manage the cost of production, the biotech and biopharmaceutical industry is constantly improving in productivity of complex therapeutic proteins. The global sale for fusion proteins is predicted to rise to $10.1 billion on a risk-adjusted basis by 2016 [8].

Proteins produced in bacterial and mammalian host cells by recombinant DNA technology often are fully or partially degraded in culture. This poses a major challenge to biotech industry [9], where efficiency and high productivity are required at lower costs. There are several reasons for low production of recombinant proteins, most of which have diagnostic and therapeutic applications. Some of these are loss of gene expression due to structural changes in the recombinant gene, loss of a gene from a plasmid, loss of plasmid, instability of plasmid (structural, segregational, multimerization) and metabolic pressure on host cells resulting in toxic metabolite accumulation and oxygen depletion. Moreover, inefficient chromosomal integration, protein aggregation, protein misfolding, improper cleavage of signal peptide and action of host cell-related proteases are common problems encountered in mammalian cell cultures for the production of recombinant proteins.

Degradation of recombinant proteins in culture is a common and serious problem [10,11]. Degradation of most proteins, including recombinant monoclonal antibodies and fusion proteins, may be attributed to host cell-derived proteases, which may be very site specific or have a broad substrate range. The most common strategies adopted to control proteolysis are engineering host cell lines by making them protease-negative or mutants for intracellular protease expression and for efficient post-translational modification and secretion. Some other methods used are genetic engineering, by mutating known protease sites in transfecting gene of interest, isolating and analyzing the type of protease secreted by CHO cells and inhibiting specifically, media optimization [12], lowering culture temperature [12,13,14], use of protease inhibitors and early product harvesting as lysis of non-viable cells in culture releases proteases which accumulate over time and degrade the desired product.

Proteolytic activity in supernatants of CHO cell cultures might be characterized using zymography or protease screening with various substrates, or by shotgun proteomics as a measure to improve the quality of recombinant proteins produced by CHO and CHO-derived cultures. In fact, the heterogeneity of recombinant glycoproteins produced in CHO culture may be attributed to modifications as a result of the action of proteases secreted by CHO. It has been earlier shown by Sandberg *et al.* that matrix metalloproteases (MMPs), including MMP3, MMP10, and MMP12, as well as their autoproteolytic smaller products, are secreted by CHO cell line during the production of recombinant factor VIII [15]. Similarly, DHFR-deficient CHO cells have been found to continuously secrete cysteine endopeptidases [16]. Serine proteases aprotinin and AEBSF (4-(2-Aminoethyl) benzene sulfonyl fluoride hydrochloride) have been shown to largely inhibit proteolytic activity in CHO cells producing gp120 protein The addition of iron citrate in CHO culture producing IFN-γ seemed to inhibit proteolysis of final product, suggesting the presence of metalloproteases in culture [17]. 

In our laboratory, a soluble anti-VEGF molecule, VEGFR1(D1–D3)-IgGFc fusion protein that was being cloned and developed in mammalian CHOK1SV GS-KO system, appeared to be undergoing clipping upon expression in culture. VEGFR1(D1–D3)-Fc is a fusion protein containing extracellular domains 1, 2 and 3 of human VEGF receptor 1 fused to Fc region of antibody IgG1. The fusion protein acts as a decoy receptor by binding to and neutralizing cellular growth factor, VEGF. This makes VEGF unavailable to its major cellular receptor VEGFR2 inhibiting downstream signaling processes ultimately leading to inhibition of cell growth, proliferation, migration, and tube formation. The clipping of the protein in culture was evident from the SDS-PAGE and Western blot analysis of the cell culture supernatants collected over all days in culture at all stages. In the present study, we have shown different ways of minimizing degradation in VEGFR1(D1–D3)-Fc fusion protein in lab scale experiments. Once proteolysis was under control, the highest yielding clone, P8D6, was taken to the roller bottle stage and, subsequently, to a bioreactor for protein production. The ligand-binding efficacy, characterization, stability and functional activity of the protein were also studied and the protein was found to be stable and functionally active.

## 2. Results

### 2.1. Generation of Fc-Fusion Protein

The VEGFR1(D1–D3)-Fc gene was generated by fusing the first three domains (D1, D2 and D3) of VEGFR1 gene from a VEGFR1 clone to the Fc (CH_2_–CH_3_) region of IgG1 of a monoclonal antibody clone already existing in our laboratory. Multiple overlap polymerase chain reactions were used to generate the fusion gene, followed by cloning the gene in mammalian expression vector, pXC17.4, and transfecting CHOK1SV GS-KO cells for generation of stable clones. The amino acid sequence of VEGFR1(D1–D3)-Fc protein is given below:

MVSYWDTGVLLCALLSCLLLTGSSSGSKLKDPELSLKGTQHIMQAGQTLHLQCRGEAAHKWSLPEMVSKESERLSITKSACGRNGKQFCSTLTLNTAQA**N**HTGFYSCKYLAVPTSKKKETESAIYIFISDTGRPFVEMYSEIPEIIHMTEGRELVIPCRVTSP**N**ITVTLKKFPLDTLIPDGKRIIWDSRKGFIIS**N**ATYKEIGLLTCEATVNGHLYKTNYLTHRQTNTIIDVQISTPRPVKLLRGHTLVL**N**CTATTPLNTRVQMTWSYPDEKNKRASVRRRIDQSNSHANIFYSVLTIDKMQNKDKGLYTCRVRSGPSFKSV**N**TSVHIYDKAFIDKTHTCPPCPAPELLGGPSVFLFPPKPKDTLMISRTPEVTCVVVDVSHEDPEVKFNWYVDGVEVHNAKTKPREEQY**N**STYRVVSVLTVLHQDWLNGKEYKCKVSNKALPAPIEKTISKAKGQPREPQVYTLPPSRDELTKNQVSLTCLVKGFYPSDIAVEWESNGQPENNYKTTPPVLDSDGSFFLYSKLTVDKSRWQQGNVFSCSVMHEALHNHYTQKSLSLSPGK

Amino acid positions:

Signal sequence (1–26)
VEGFR1 domain D1 (32–123)
VEGFR1 domain D2 (151–214)
VEGFR1 domain D3 (230–327)
IgG1 Fc CH2 domain (342–454)
IgG1 Fc CH3 domain (455–561)

There are six sites for *N*-glycan attachment in the molecule at N (asp) shown in bold and underlined in the text, at amino acid positions:

VEGFR1(D1): 100
VEGFR1(D2): 164, 196
VEGFR1(D3): 251, 323
IgG1-Fc (CH_2_): 411

### 2.2. Protein Clipping in Different Host Cells

**Figure 1:**
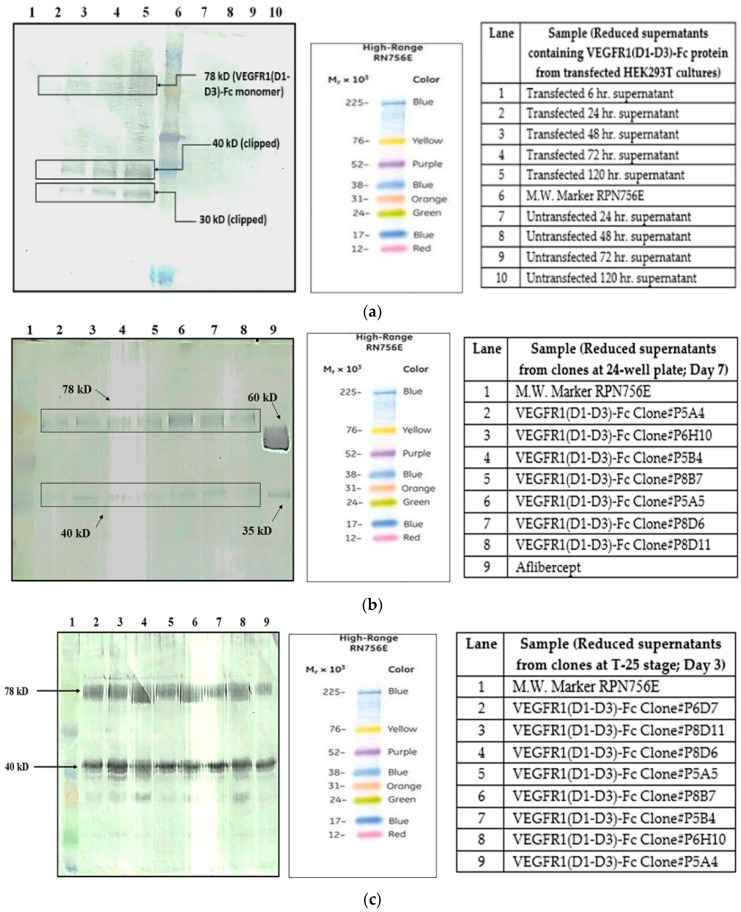

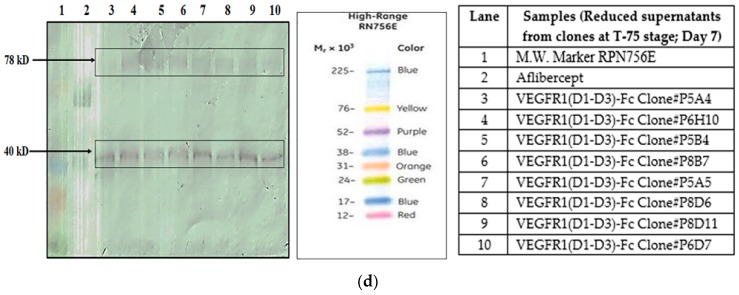
(**a**) Western Blot of supernatants of transfected HEK293T cells expressing VEGFR1(D1-D3)-Fc protein at different time points in 24-well plate; (**b**) Western Blot for VEGFR1-Fc detection in supernatants of transfected CHOK1SV GS-KO clones at 24-well plate stage of clone development; (**c**) Western Blot of supernatants of clones at T-25 stage for VEGFR1(D1–D3)-Fc protein expression analysis; (**d**) Western Blot of supernatants of clones at T-75 stage for VEGFR1(D1–D3)-Fc protein expression analysis.

Supernatants from transfected HEK293T cells in 24-well plate were collected after different time points (6, 24, 48, 72 and 120 h) and protein expression checked by Western blot under reducing conditions (Figure 1a). Similarly, supernatants from multiple clones of CHOK1SV GS-KO at various clone development stages were analyzed by Western blot for presence of expressed VEGFR1(D1–D3)-Fc protein (Figure 1b–d).

It is clearly evident from the Western blots performed under reducing conditions that VEGFR1 (D1-D3)-Fc is being clipped immediately after expression from both host cell lines. Cleavage of this Fc protein is probably independent of host cell.

### 2.3. Clone Specific Protein Degradation

**Figure 2:**
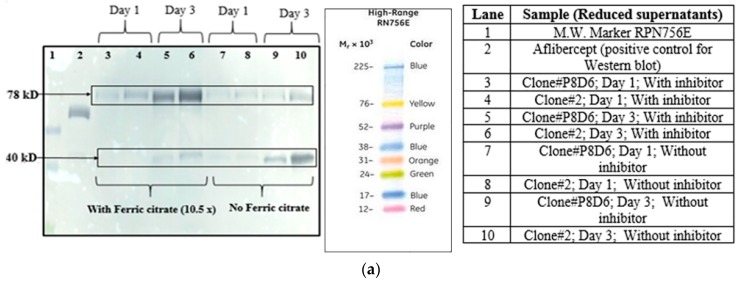

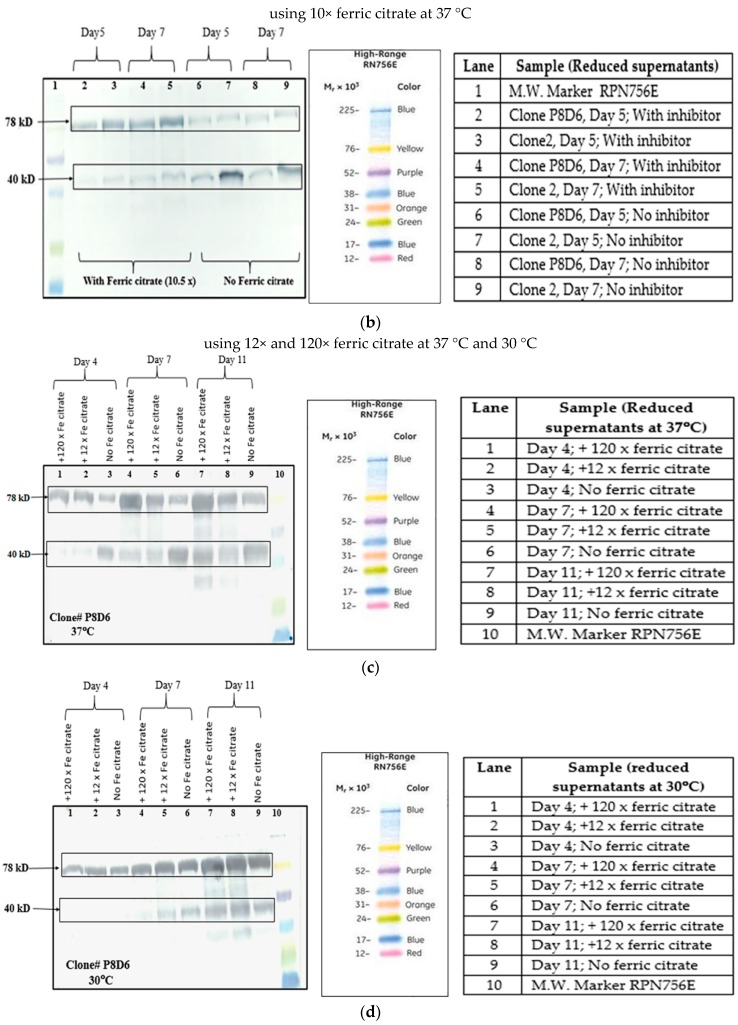
(**a**) Western Blot for VEGFR1(D1–D3)-Fc expression with and without 10× ferric citrate in culture supernatant for two different clones; (**b**) Western Blot for VEGFR1(D1-D3)-Fc expression with and without 10× ferric citrate in culture; (**c**) effect of ferric citrate on reduction of proteolysis in VEGFR1(D1-D3)-Fc at 37 °C; (**d**) effect of ferric citrate on reduction of proteolysis in VEGFR1(D1-D3)-Fc at 30 °C.

Two different CHOK1SV GS-KO clones for VEGFR1(D1–D3)-Fc, clone P8D6 and clone 2 were cultured in T-25 flasks in CDCHO media at 37 °C and 5% CO_2_ in presence and absence of ferric citrate as a prospective proteolysis inhibitor. Protein degradation pattern was not identical in the clones suggesting that proteolysis is clone specific (Figure 2a). Lanes 9 and 10 show different profiles of two clones on day 3 growing at 37 °C in culture medium containing no proteolytic inhibitors. The difference in the degree of proteolysis is that different clones may be attributed to their protein expressing ability as well.

### 2.4. Protease Inhibition Assays

#### 2.4.1. Lowering of Culture Temperature

**Figure 3:**
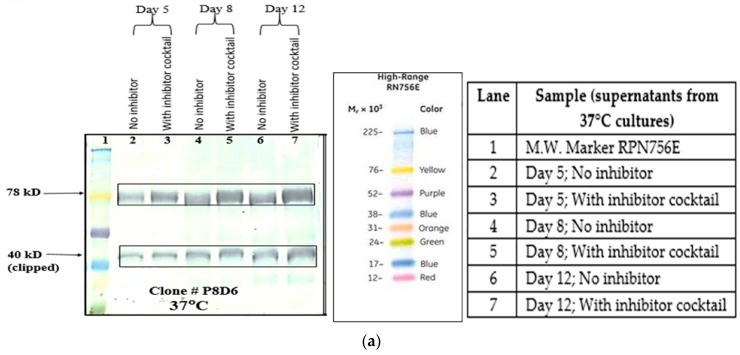

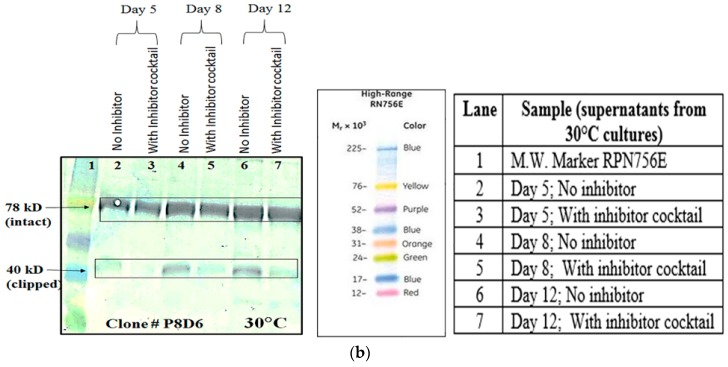
(**a**) Effect of protease inhibitor cocktail on reduction of proteolysis in VEGFR1-Fc at 37 °C; (**b**) effect of protease inhibitor cocktail on reduction of proteolysis in VEGFR1(D1–D3)-Fc at 30 °C.

It was observed that maintaining the culture at 30 °C, significantly reduces degradation of the protein expressed, even though productivity and cell growth decreases to a large extent. The proteolysis inhibitory effect of lower culture temperature is further enhanced by the addition of inhibitors of protein degradation to the cell culture. Culturing cells at a lower temperature (30 °C) effectively reduces proteolysis to a large extent, as is evident from Western blots (Figure 2c,d). Lane number 2 from blots clearly show that the clipped product of VEGFR1(D1–D3)-Fc (40 kDa protein) at 37 °C is more than the intact protein (78 kDa), whereas, at 30 °C, the scenario is reversed. Upon inclusion of protease inhibitors at low temperatures, protein degradation is further arrested (Figure 3a,b). Culturing cells at a lower temperature also prolongs cell viability over time, even though total protein productivity is compromised. Cell viability at day 8 in culture at 30 °C is almost 90%, whereas it is about 70% for culture grown at 37 °C (Supplementary Figure S1). This degradation process progresses with a number of days in culture.

#### 2.4.2. Addition of Ferric Citrate

Two VEGFR1-Fc clones (clones 2 and P8D6) were cultured in the presence of 10× ferric citrate in CDCHO media at 37 °C and 5% CO_2_. Western blots were performed with culture supernatants collected on different days and checked for degradation profile of protein in presence and absence of inhibitor for both clones (Figure 2b). The effect of a higher concentration of ferric citrate (120×) and at a lower culture temperature was even more pronounced (Lane 10; Figure 2c,d).

#### 2.4.3. Addition of Protease Inhibitor Cocktail

The P8D6 cultures were grown in the presence of protease inhibitor cocktail at 37 and 30 °C in order to study the individual as well as additive effect of cocktail inhibitors with low temperature, if any, on proteolysis inhibition. It was observed, as of day 12, that culturing cells alone at 30 °C definitely reduced protein clipping relative to 37 °C (Lane 6; Figure 3a,b) and addition of protease inhibitor cocktail further prevented the degradation process (Lane 7; Figure 3a,b).

### 2.5. Scale-up Production of VEGFR1(D1–D3)-Fc in Bioreactor

**Figure 4:**
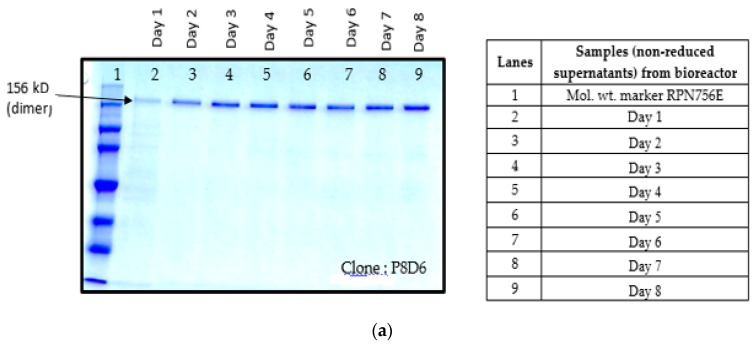

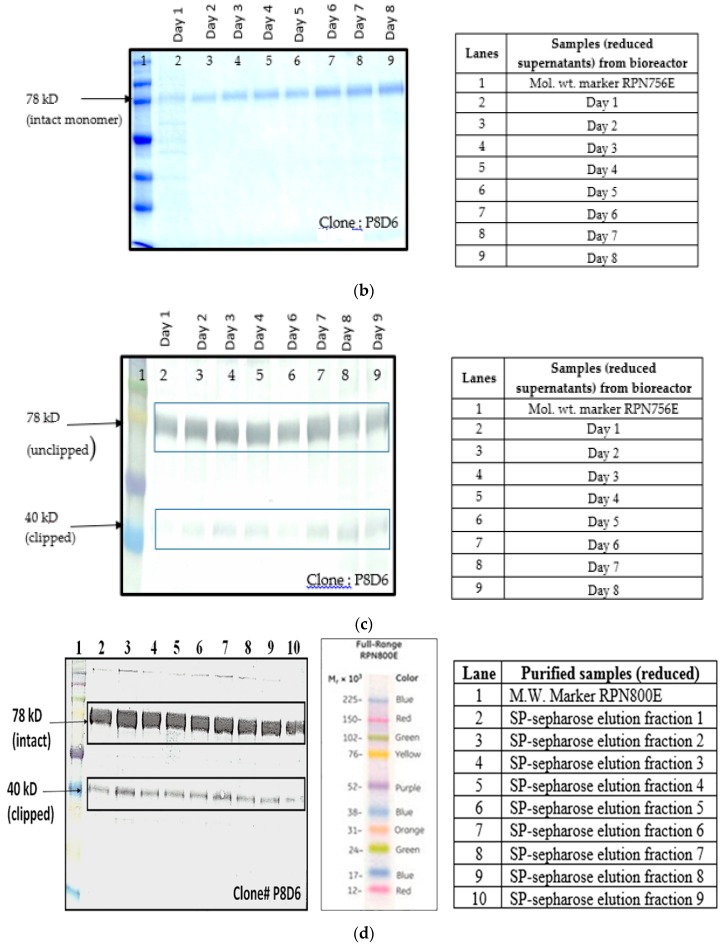
(**a**) SDS-PAGE of supernatant samples of bioreactor culture on various days (non-reduced); (**b**) SDS-PAGE of supernatant samples of bioreactor culture on various days (reduced); (**c**) Western blot of samples from bioreactor (reduced); (**d**) Western Blot of VEGFR1(D1–D3)-Fc purified fractions from SP-Sepharose cation exchange chromatography (reduced samples).

Once it was observed that proteolysis of the molecule can be reduced at a low culture temperature, addition of ferric citrate or protease inhibitor cocktail and harvesting at an earlier time, the highest VEGFR1(D1–D3)-Fc yielding clone, P8D6 was cultured at 30 °C in a 4-L bioreactor in fed-batch mode and harvested on day 8. SDS-PAGE and Western blots were used to analyze supernatants for profiling of expressed protein (Figure 4a–c). The protein was purified using Protein A affinity chromatography followed by SP-sepharose strong cation exchange chromatography. The purified fractions eluted from the ion-exchange chromatographic column were analyzed by Western blot (Figure 4d) before pooling and concentrating in the appropriate buffer for further analyses. It is evident from SDS-PAGE and Western blot of culture supernatants, and of purified protein, that the degree of proteolysis in culture has been reduced substantially, with the majority of the protein present in the intact form.

### 2.6. RP-HPLC

**Figure 5:**
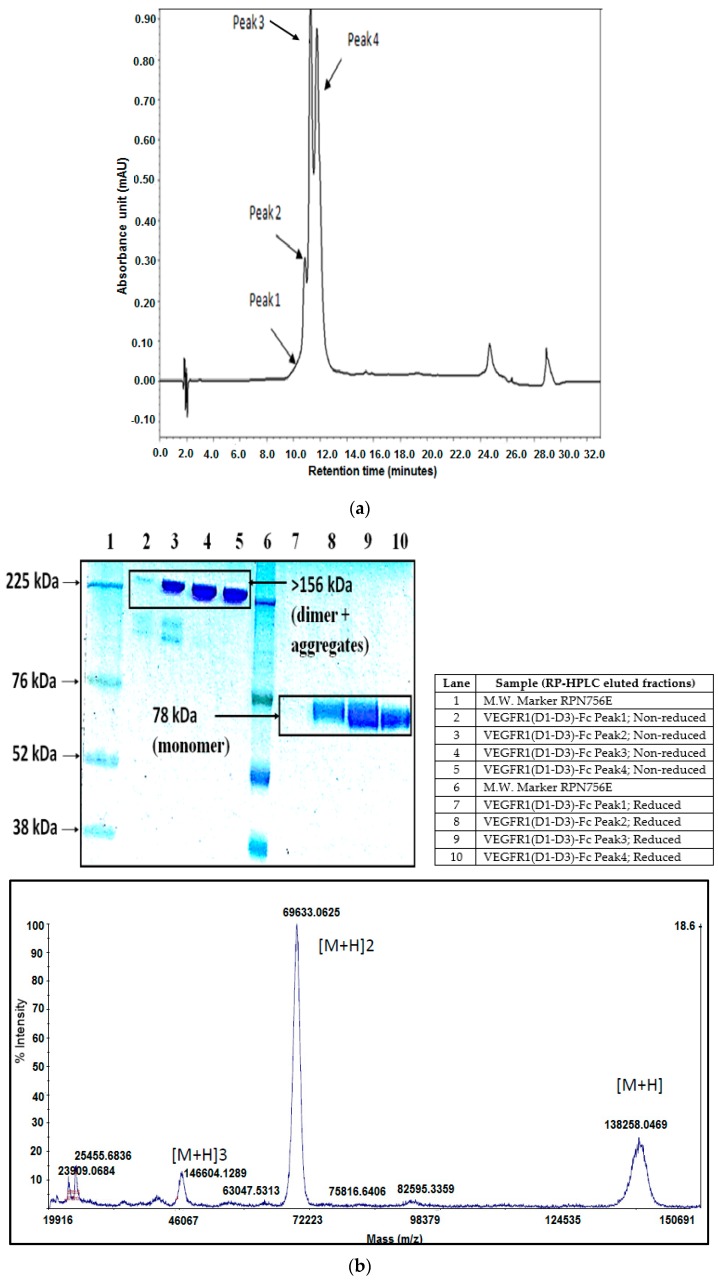

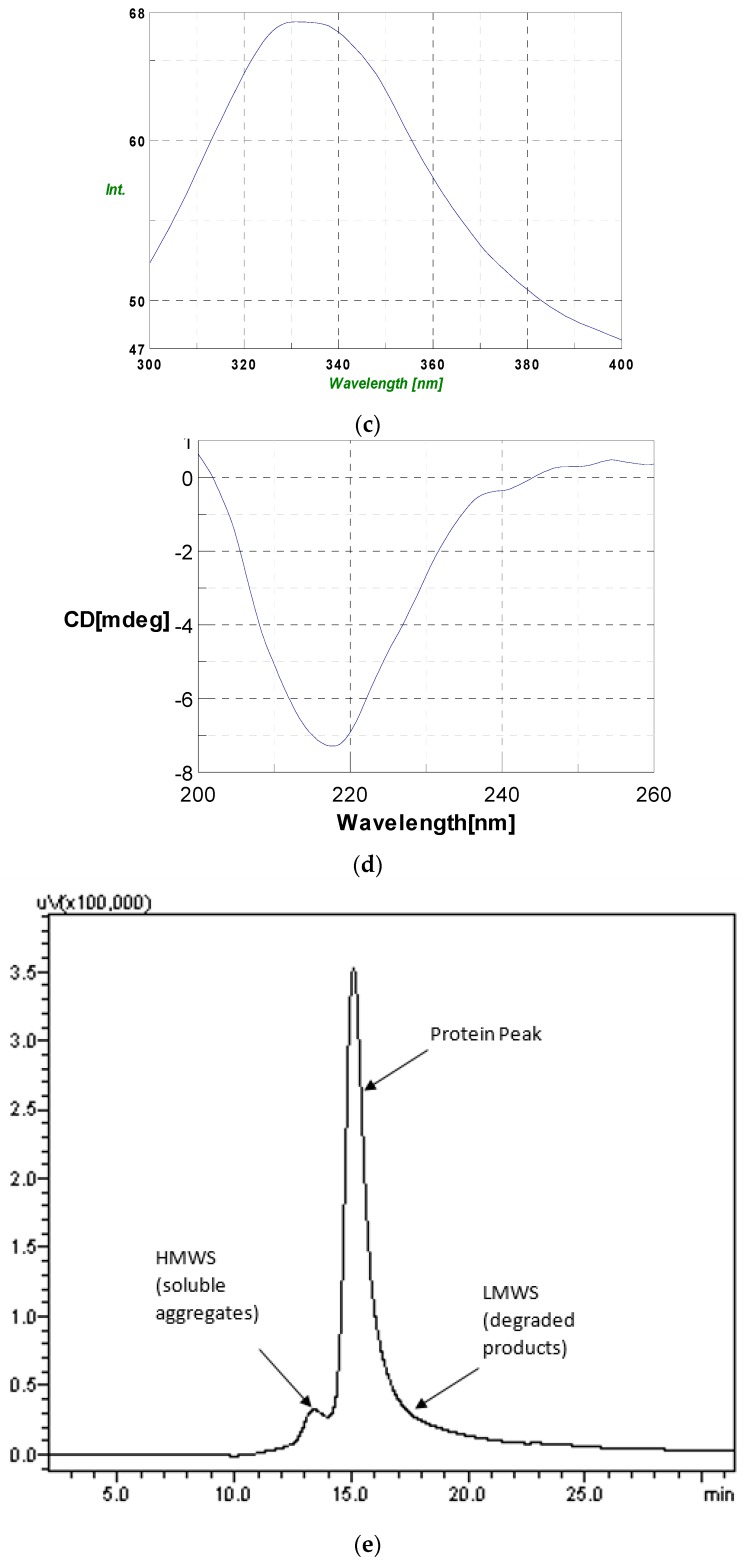

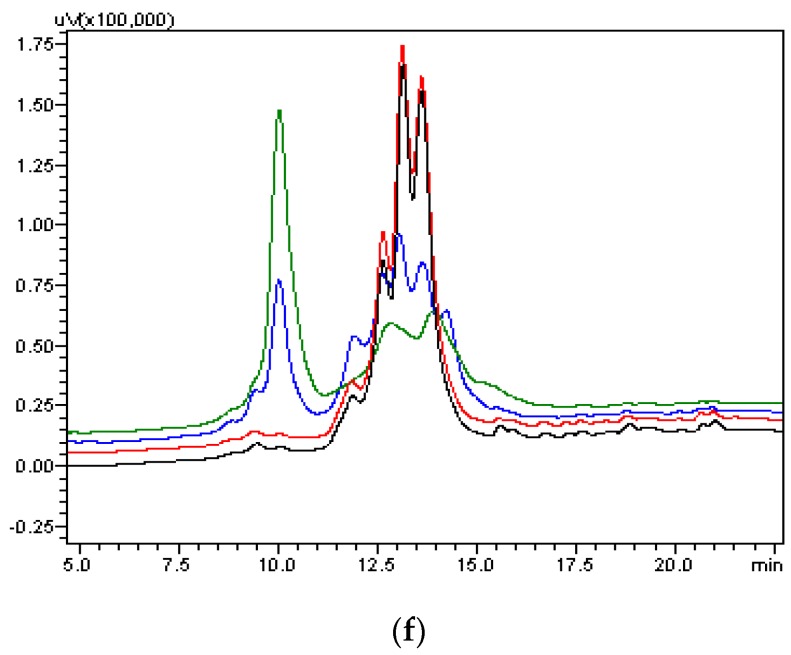
(**a**) VEGFR1(D1–D3)-Fc profiling using analytical RP-HPLC; (**b**) MALDI TOF/TOF MS spectra for intact mass analysis for VEGFR1-Fc peak #3; (**c**) fluorescence spectroscopic data for VEGFR1(D1–D3)-Fc; (**d**) far UV CD data for VEGFR1(D1-D3)-Fc protein; (**e**) SEC data for VEGFR1(D1–D3)-Fc; (**f**) VEGFR1(D1–D3)-Fc protein stability at 6 months by RP-HPLC. Black: −70 °C; Red: −20 °C; Blue: 2–8 °C; Green: 25 °C. RP-HPCL data implies protein is stable at 6 months at −70 °C and also to a great extent at −20 °C.

The purified protein was concentrated to 10 µg using Amicon^®^Ultra-4, Ultracel 30k filter (Millipore; catalogue # UFC803096; Lot # R6SN15198, Billerica, MA, USA) before being loaded onto an analytical RP-HPLC column (Figure 5a). Four peaks were eluted from the run and individually collected. Peak 3’s fraction was dried under vacuum and characterized by mass spectrometry.

### 2.7. Mass Spectroscopy

Mass spectroscopy was performed (at Centre for Cellular and Molecular Platforms (C-CAMP), Bangalore, India) to measure the intact mass/molecular weight of the protein [18]. MALDI-TOF MS spectrum of Peak 3’s protein shows three different charge states of the sample (Figure 5b). The most abundant glycosylation isoform of the protein shows mass by charge range of 68,500 to 71,500 Da; *Z* = 2. This implies a mass in the range of 137,000 to 143,000 Da. The VEGFR1(D1–D3)-Fc is a dimeric glycoprotein having six *N*-linked glycosylation sites in a monomer, each contributing to 2.5 kDa or a total of 30 kDa. This would make the fully glycosylated molecule have a mass of 156 kDa. Non-glycosylated form of the protein has a molecular weight of 126 kDa. Hence, the observed mass of the peak sample analyzed by mass spectrometry (137 to 143 kDa) implies that the protein is a partially glycosylated form of VEGFR1(D1–D3)-Fc.

### 2.8. Fluorescence Spectroscopy

Total fluorescence of a protein is a mixture of the fluorescence from individual aromatic residues. The two amino acids that fluoresce are tryptophan (excitation maximum at ~280 nm) and tyrosine (excitation maximum at ~276 nm). Tryptophan (Trp), with a much larger extinction coefficient and fluorescence quantum yield, is the most prominent emitter [19]. In general, the more buried these residues are within the protein the less fluorescence quenching the more they will experience, including a reduced solvent induced fluorescence quenching. Fluorescence intensity of a protein can either increase or decrease during folding, with tryptophan fluorescence often used as a means of determining both stability and folding of a protein.

The fluorescence data indicates reasonable tertiary structure of the protein as the chromophores show strong emission at 330 nm. Both Trp and tyrosine (Tyr) have a wavelength of maximum absorption at around 280 nm, but at 295 nm, only Trp gets excited and not the weaker Tyr. Emission peak of Trp ranges from 300 to 350 nm depending on the polarity of the solvent. In Figure 5c, wavelength excitation maximum is ~330 nm, which shows good folding of the protein suggesting tryptophan moieties deeply buried within the protein without getting exposed to local environment consisting of solvent, denaturants, surfactants and presence of nearby protonated amino acid residues, thus minimizing fluorescence quenching. Overall, the fluorescence data indicate intact tertiary structure of VEGFR1(D1–D3)-Fc.

### 2.9. Circular Dichroism

CD is a valuable spectroscopy technique for examining the secondary structure and protein folding in a wide range of wavelengths [20]. It also studies the conformational stability of a protein in solution. One of the strengths of CD is that various aspects of protein structure can be determined. In the far UV, the peptide bond is the principal absorbing group; studies in this region can give information on the secondary structure. In the near UV, the aromatic amino acid side chains (Phe, Tyr, Trp) absorb in the range 250 to 290 nm. The tertiary folding of the polypeptide chain can place these side chains in chiral environments, thus, giving rise to CD spectra, which can serve as characteristic fingerprints of the native structure.

The far-UV CD data clearly show good secondary structures. (Figure 5d) The raw data was deconvoluted using BeStSel (beta structure selector) online platform [21] to assess the percentage of secondary structure elements predicted α-helix at 9.5% and β-strand at 33% (antiparallel: 33% and parallel: 33.5%).

### 2.10. Size Exclusion Chromatography

SEC data implies the presence of 87.22% of an intact protein, along with 7.78% of high molecular weight aggregates and about 5% low molecular weight impurities in the form of degraded products.

### 2.11. Protein Stability Determination

Stability of protein samples stored at different temperature conditions (−70, −20, 2–8, 25, and 40 °C) was determined at various time points (0 day, 15 days, 1 month, 3 months and 6 months) by SDS-PAGE, Western blot and RP-HPLC. Data after 6 months show that the protein is stable at −70 °C and −20 °C, whereas it starts progressively degrading at higher temperatures beyond this time (Figure 5f). Since the SDS-PAGE and RP-HPLC profiles after the second month suggest complete degradation of the protein at 40 °C, this temperature was not studied for further time points. With the increase in storage temperature, the amount of cleaved products represented as low molecular weight impurities increases considerably.

### 2.12. Ligand-Binding Affinity of VEGFR1(D1–D3)-Fc by ELISA

**Figure 6:**
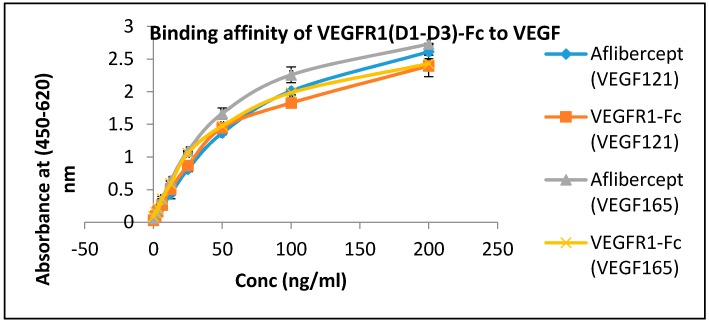
Binding efficiency comparison of VEGFR1(D1–D3)-Fc and aflibercept to VEGF isoforms by ELISA.

The affinity of VEGFR1(D1–D3)-Fc protein towards its ligands, VEGF_165_ and VEGF_121_ was checked using ELISA and compared to the affinity of another fusion protein, VEGF Trap_R1R2_ towards the same ligands (Figure 6). It was observed that VEGFR1(D1–D3)-Fc exhibits a strong affinity towards its natural ligands, and its affinity towards both the VEGF ligands was almost comparable to that of a known decoy receptor trap, aflibercept.

### 2.13. Inhibition of Endothelial Cell Proliferation by VEGFR1(D1–D3)-Fc

**Figure 7:**
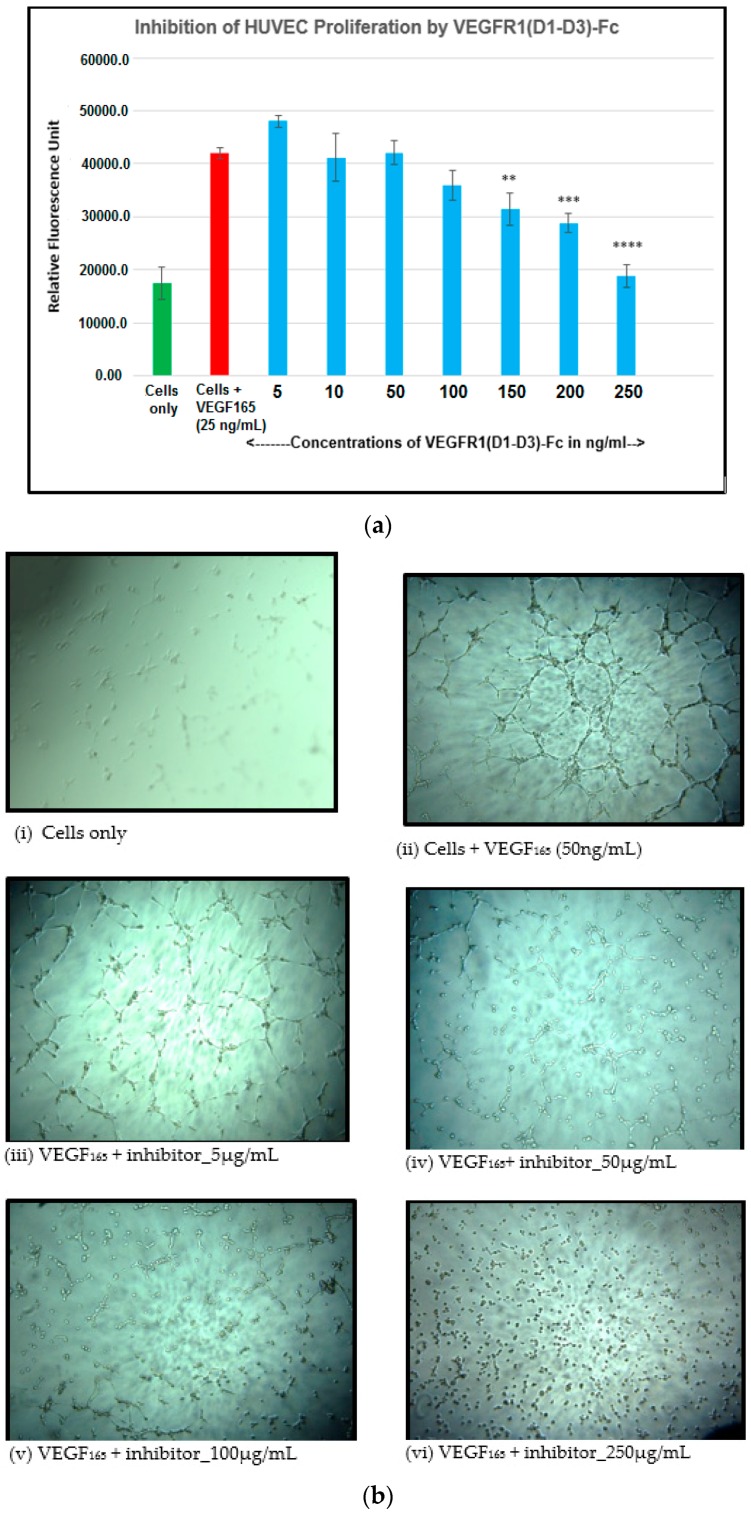

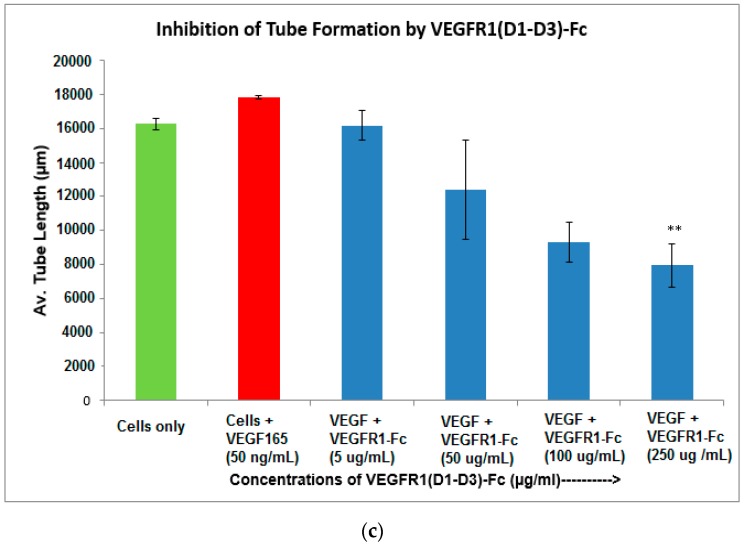
(**a**) Endothelial cell proliferation assay of VEGFR1(D1–D3)-Fc; **, *p* ≤ 0.01, ***, *p* ≤ 0.001; ****, *p* ≤ 0.0001; (**b**) images of endothelial tube formation assay for VEGFR1(D1–D3-Fc. Images were viewed under magnification 10×; Scale bar = 200 µM. **b**(**i**) HUVEC (5000 cells) only plated on matrigel; (**ii**) HUVEC + VEGF_165_ (50 ng/mL) in absence of inhibitor; (**iii**) HUVEC + VEGF_165_ + VEGFR1(D1–D3)-Fc (5 μg/mL); (**iv**) HUVEC+ VEGF_165_+ VEGFR1(D1-D3)-Fc (50 μg/mL); (**v**) HUVEC + VEGF_165_ + VEGFR1(D1-D3)-Fc (100 μg/mL); (**vi**) HUVEC + VEGF_165_ + VEGFR1(D1–D3)-Fc (250 μg/mL; (**c**) Endothelial tube formation assay of VEGFR1(D1–D3)-Fc; **, *p* ≤ 0.01.

Inhibition of proliferation was demonstrated in a HUVEC proliferation assay using Alamar Blue fluorescence as indicator. Viability was assessed by reduction of Alamar Blue, which was measured spectrophotometrically at 544/590 nm. VEGFR1(D1–D3)-Fc shows reduction of VEGF-stimulated cell proliferation with increasing concentration of the inhibitor (Figure 7a).

### 2.14. Inhibition of Endothelial Tube Formation by VEGFR1(D1-D3)-Fc

VEGFR1(D1–D3)-Fc protein was used to check its inhibitory activity on network formation by endothelial cells. Images were taken with a digital camera fitted to an inverted microscope under 5× magnification (Figure 7b,c).

## 3. Discussion

We investigated the effects of different culture parameters like culture temperature, culture duration, and chemical inhibitor supplementation on protease activity in CHOK1SV GS-KO culture expressing recombinant Fc fusion protein, VEGFR1(D1–D3)-Fc.

VEGFR1(D1–D3)-Fc protein was expressed in two host cells, HEK293T and CHOK1SV GS-KO. Recombinant protein expression levels were checked using Western Blot for VEGFR1-Fc in the cell culture supernatants of transfected cultures. Protein expressed from both mammalian host cell lines was clipped as detected by Western blots. Hence, host specific cleavage of the recombinant protein is not evident from this study. Further, upon checking degradation pattern of the same protein in two different CHOK1SV GS-KO clones (clone#P8D6 and clone#2), it was observed that the degradation patterns were similar in both clones. However, the amount of degradation was varying which might be dependent on the levels of productivity of the clones. This clone-to-clone variation, with respect to the susceptibility of expressed proteins to proteases, might be because CHO is not a clonal cell line due to the dynamic nature of its functional genome and potential to evolve [22]. Cell viability and cell count patterns for both clones are different over days in culture (Supplementary Figures S2 and S3). Several studies have shown that the degree of glycosylation of a glycosylated protein is directly related to the susceptibility to proteases with the presence of more glycan moieties, reducing accessibility of proteases to proteolytic cleavage sites in proteins [10,23]

Proteolysis can also occur as a result of lysis of non-viable cells, which release proteases in the medium, which in turn is responsible for the degradation of the product. One way to minimize this process is by reducing the clumping of cells in culture. When clumping or aggregation occurs, the overall surface area exposed to available medium is reduced resulting in reduced growth rate, cell densities, and productivity. Additionally, cells in the middle of a large clump are more prone to the stress of limiting nutrients and toxic build-up leading to programmed cell death/apoptosis, followed by lysis and protease release. Iron is essential for cell respiration and metabolism and protects cells from oxidative damage. Ferric citrate is a soluble iron source for serum-free medium. It has been previously shown to inhibit metalloproteases, probably by blocking zinc atoms in the catalytic domain of the enzymes [17]. Rosenmund *et al.* have also shown that, in physiological conditions, serine proteases are susceptible to nontransferrin-bound Fe^3+^ [24]. Upon addition of ferric citrate to cell culture at a concentration of 10×, it was observed that cell count and viability for clone P8D6, increased with days in culture at 37 °C as compared to control (Supplementary Figure S4). The addition of protease inhibitor cocktail (serine and cysteine proteases inhibitors) to culture, increased cell viability with increased number of days in culture, as compared to control without inhibitor (25% *vs.* 76% on day 12).

It has been already reported that lowering of culture incubation temperatures from 37 to 34, 33, or even 28 °C, increases the product quality in terms of reducing clipping of protein [25,26]. At a culture temperature of 30 °C, although growth of cells was suppressed, cell viability remained high for a prolonged period of time.

As part of validation, a large-scale production of VEGFR1-Fc was carried out in a 4-L bioreactor in a fed-batch mode. VEGFR1(D2–D3)-Fc producing clone P8D6 was cultured in a roller bottle at 37 °C until a one-liter inoculum was ready to be seeded in a bioreactor containing 4 L of CDCHO medium. During inoculum preparation in roller bottles, ferric citrate (12×) was added to the culture medium in an attempt to have a minimum amount of carry over clipped species in the bioreactor. No additives in the form of protease inhibitors were added to the bioreactor, as it would have later implications from a regulatory point of view during large-scale production. Under fed-batch mode of culture, the cells were grown in the bioreactor at 30 °C for 8 days, and supernatants were analyzed every day using SDS-PAGE and Western blots. Reduction in the levels of protein degradation was observed. Since the stability of the expressed Fc-fusion protein appeared to decrease with time in culture, the culture was harvested at an earlier time point to ensure minimum proteolysis. Protein A chromatography of VEGFR1(D1–D3)-Fc from harvest resulted in a purified protein, which contained clipped species along with the intact protein. The final purified protein, upon analysis by size exclusion chromatography, showed a purity of above 87%. Degraded low molecular weight species constituted 5% of total protein and about 8% formed high molecular weight aggregates. However, in spite of cleaved nature of the protein, ELISA studies confirmed significant binding affinity towards ligands, VEGF_165_ and VEGF_121_ compared to a known VEGF receptor-Fc fusion protein, aflibercept. Analytical RP-HPLC data for the purified protein showed four peaks, of which the major peak was analyzed by mass spectrometry and found to be one of the glycosylated forms of VEGFR1(D1–D3)-Fc. Circular dichroism and fluorescence data suggested that the protein was in a properly folded form with intact secondary and tertiary structures and overall three-dimensional stability. The secondary structure consisted of about 50% alpha helix and 10% beta sheets for the protein as predicted by CD. The intrinsic fluorescence of the protein results in strong chromophore emission at 330 nm and minimal fluorescence quenching, which predicts properly the folded stable tertiary structure of the protein. Stability studies of the protein suggested that the protein is stable at −20 °C and −70 °C for a six month period, as is evident from RP-HPLC profiles at various time points and at different temperature conditions. The protein was also shown to be biologically active, as was evident from its ability to inhibit both endothelial cell proliferation, as well as endothelial tube formation in HUVEC *in vitro* assays.

## 4. Materials and Methods 

### 4.1. Cell Culture

CHOK1SV GS-KO (Lonza Biologics, Slough, Berkshire, UK) was the mammalian cell line used for producing VEGFR1(D1–D3)-Fc protein in culture. Cells were cultured in CDCHO proprietary chemically defined medium (Gibco, Santa Clara, CA, USA). For stable transfection, cells and 50 µg of linearized plasmid (pXC17.4 vector containing the gene of interest VEGFR1-Fc) were transferred to a 0.4 cm Biorad cuvette and electroporated using a Bio-Rad GenePulser instrument (Hercules, CA, USA) set at 0.298 kV and 900 μF with a time constant of 18.6 ms. All stable recombinant protein-producing clones using glutamine synthetase (GS) knock out (KO) system was cultured in plain CDCHO medium without glutamine or methionine sulfoximine (MSX). Clones were passaged from 96-well plate to 24-well plate followed by sequential transfer to a T-25, T-75, T-175 flask and a roller bottle at a density of 2 × 10^5^ cells/mL in each sub-culturing stage. 

#### 4.1.1. Different Host Cells

It is known that one of the reasons for clipping of Fc fusion proteins is a result of the action of proteases secreted by host cells harboring the gene of interest [5]. In order to find out whether proteolysis is dependent on the type of host cell, VEGFR1(D1–D3)-Fc recombinant gene was transfected in two different mammalian cell lines, HEK293T, and CHOK1SV GS-KO. HEK293T cells were transiently transfected with a plasmid containing VEGFR1(D1–D3)-Fc using lipofectamine™ 2000 (Life Technologies, Carlsbad, CA, USA) as the transfection agent, whereas CHOK1SV GS-KO cells were stably transfected by electroporation. VEGFR1(D1–D3)-Fc yielding CHOK1SV GS-KO clones were propagated through various stages of development by passaging from 96-well plate through a 24-well plate, T-25, T-75, T-175 flasks and a roller bottle. Western blot analyses were performed at every stage of clone development in order to check for expression levels of the protein under both reducing and non-reducing conditions (Figure 1a–d).

#### 4.1.2. Different Clones Expressing Same Protein

Two different clones (clone P8D6 and clone 2) both expressing VEGFR1-Fc protein, were cultured in CDCHO media at 37 °C. Expression of protein in cell culture supernatant for both clones on different days in culture was compared by Western Blot (Figure 2a). The clones were cultured in the presence and absence of anti-clumping agent ferric citrate as a proteolysis inhibitor. 

### 4.2. SDS-PAGE and Western Blot

Culture supernatants at all stages of clone development (24 well plates, T-25, T-75, T-175 and roller bottle, bioreactor) were analyzed for expression of VEGFR1(D1–D3)-Fc protein by Western Blot under reducing and non-reducing conditions. Samples were resolved on 8%–10% polyacrylamide gels in parallel to molecular weight marker (high range rainbow marker, Amersham, GE Healthcare). Samples were reduced using β-mercaptoethanol and heat-denaturation prior to loading. Following electrophoresis, gels were transferred to nitrocellulose membrane by passive blotting, and protein was detected using HRP-conjugated goat anti-human IgG-Fc-γ specific polyclonal antibody (Catalog #109-036-098; Jackson Immuno Research Labs Inc., West Grove, PA, USA) and developed by DAB-urea (SigmaFast™ DAB (3,3′-Diaminobenzidine tetrahydrochloride) with Metal Enhancer-Urea hydrogen peroxide tablet, St. Louis, MO, USA) substrate solution.

### 4.3. Protease Inhibition Assays

#### 4.3.1. Temperature

CHOK1SV GSKO culture expressing VEGFR1(D1–D3)-Fc was incubated at 37 and 30 °C in T-175 flasks in a 5% CO_2_ incubator. Degree of proteolysis was monitored until day 11 in culture using Western Blot. The effect of low temperature (30 °C) in presence of protease inhibitors was also studied at the T-175 stage. The effect of temperature on cell growth was assessed by cell counting and cell viability check and protein expression levels were analyzed by Western Blot of culture supernatants.

#### 4.3.2. Ferric Citrate

Ferric citrate is used as an anti-clumping agent for reducing CHO cell aggregation in culture. It is also known to inhibit metalloproteases and serine proteases present in the culture [24]. Ferric citrate (anti-clumping agent B, Lonza, Slough, Berkshire, UK) at concentrations of 10×, 12× and 120× were used in CDCHO media as an inhibitor of proteolysis, while culturing cells at both 30 and 37 °C. It is a proprietary formulation and comes as a 1000× concentration, which we have diluted according to our experimental needs. Cell counts on regular intervals were taken to check for cell growth and productivity was analyzed by Western Blot of culture supernatants. The effect of 10× ferric citrate was first studied at 37 °C at the T-25 flask stage over a period of 10 days in culture for two different VEGFR1-Fc clones. Next, the effect of ferric citrate at concentrations of 12× and 120× was studied at both temperatures (37 and 30 °C) in T-175 flasks over a period of 11 days.

#### 4.3.3. Protease Inhibitor Cocktail

Effect of protease inhibitor cocktail (cOmplete EDTA-free protease inhibitor cocktail tablet, Roche, catalog# 11 873 580 001) on VEGFR1(D1-D3)-Fc protein clipping was studied at two incubation temperatures of 37 and 30 °C. CHOK1SV GSKO clones were seeded at a density of 2 × 10^5^ cells/mL in T-175 flasks and grown for 12 days in the presence of inhibitors, which completely inhibits serine and cysteine proteases. Cell counts and viability were checked at regular time points and inhibition of protein clipping was analyzed using Western Blot.

### 4.4. RP-HPLC

The highest protein-yielding clone was cultured in 4-liter fed-batch mode and purified by Protein A affinity chromatography and strong cation exchange chromatography followed by analysis of purified protein by RP-HPLC. RP-HPLC was performed with elution fractions on an analytical C5 column with dimensions of 150 mm × 4.6 mm (Discovery Bio Wide, Supelco; Pore 5 µm; Catalog # 568422-U, Bellefonte, PA, USA) using mobile phase buffer A as 0.1% TFA in water and buffer B as 0.1% TFA in 90% acetonitrile in water. The column temperature was 60 °C and a flow rate of 1 mL per min was used to resolve proteins under the non-denaturing condition and detected by a two point UV detector at 214 nm.

### 4.5. Mass Spectrometry

Peak fractions obtained from RP-HPLC run were collected separately. The major peak, peak 3 was dried under vacuum and sent to Centre for Cellular and Molecular Platforms (C-CAMP), Bangalore, for the intact mass analysis. Myoglobin was used for calibration. The samples were analyzed on ABSCIEX MALDI-TOF 5800 (Framingham, MA, USA). All the samples are desalted and buffer exchanged in 50 mM ammonium bicarbonate of pH 7.5 by centrifugal ultrafiltration (Amicon Ultra-0.5 mL, 10 K MWCO, 14,000 g, 15 min, 4C, Billerica, MA, USA). Five microliters of 10 mg/mL of sinapinic acid was mixed with 5 µL of 1 mg/mL sample. One microliter of the sample was spotted on the plate and after drying the sample was used for MALDI-TOF analysis.

### 4.6. Fluorescence Spectroscopy

Purified protein was diluted to 1:3 times with 5 mM phosphate buffer, pH 7.0, making a final volume of 800 μL. The protein was analyzed on FP-8000 (Jasco, Easton, MD, USA). 10 µM protein was used for analysis using a path length of 10 mm.

### 4.7. Circular Dichroism

Stability and integrity of protein was analyzed by CD on J-710 (Jasco). The protein sample was diluted to 1:1 with 400 μL of 5 mM phosphate buffer pH 7.0. For data acquisition, a path length and data pitch of 1 nm each was used, with a bandwidth of 2 nm, response time of 4 s and scanning speed of 50 nm/min for 2 accumulations. For far UV CD spectrum, 5 µM of protein was analyzed.

### 4.8. Size Exclusion Chromatography

The overall molecular size and the presence of aggregated species and low molecular weight impurities were determined by Size-Exclusion HPLC analysis. SEC-HPLC chromatograms of VEGFR1 (D1–D3)-Fc are shown in the Figure 5e. An analytical gel filtration chromatographic column, TSK column was used for testing: Tosoh-Gel, G-3000 SWXL (TOSOH; catalogue number 08541, Tokyo, Japan); 7.8 mm internal diameter; 30 cm length; 5 micron particle size; 250° pore size. A total of 15 μg of protein was injected onto the column. Mobile phase used was 20 mM phosphate buffer containing 400 mM sodium chloride, pH 7.0. Flow rate was 0.5 mL/min a run time was 35 min with a UV detector (Tosoh, Tokyo, Japan) at 214 nm wavelength.

### 4.9. Protein Stability Determination

To determine the stability of VEGFR1(D1–D3)-Fc *in vitro*, purified VEGFR1(D1–D3)-Fc protein (1 mg) was aliquoted in glass vials (1 mL per vial) and kept in stability chambers of varying temperature conditions (−70 °C, −20 °C, 2–8 °C, 25 °C and 40 °C) and analyzed at different time points (0 day, 15 days, 1 month, 2 months, 3 months and 6 months) using SDS-PAGE, Western blotting and RP-HPLC profiling.

### 4.10. Detection of Ligand-Binding Affinity

An ELISA-based method was used to determine the binding affinity of VEGFR1(D1–D3)-Fc to its common physiological VEGF ligands, isoforms VEGF121 and VEGF_165_. A micro-titer plate (Nunc maxisorp) was coated with VEGF_165_ (conc. 3.3 mg/mL) and VEGF121 (conc. 0.9 mg/mL) at a concentration of 250 ng/100 μL/well and coating was allowed to take place at 4 °C, overnight, following which the plate was washed one time with 1× PBS (300 µL/well). Contents from the plate were flicked off and 300 µL of 1% BSA in 1X PBST was added to each well and blocking allowed to take place for one hour at 37 °C. Dilutions of test sample {VEGFR1(D1–D3)-Fc} were prepared in 0.1% BSA in 1× PBST (0.05% PBST) in the range of 200 to 1.5 ng/mL. Aflibercept, used as a positive control, was similarly diluted. The plate was washed three times with 300 µL of wash buffer (0.05% PBST) per well. Standard dilutions and test samples were added to respective wells of the plate and plate incubated at 37 °C for 1 h. The plate was again washed three times with wash buffer as before. Next, goat anti-human IgG-Fc (Jackson; Cat#109-036-098) specific horse radish peroxidase labeled secondary antibody at 1:40,000 dilutions (in 0.1% BSA in 1× PBST) were added to each well and incubated at 370C for one hour. This was followed by three washes with 0.05% PBST. Finally 100 µL of 1× TMB substrate were added to each well and plate incubated in dark for 30 min before stopping the reaction by addition of 100 µL 1 N H_2_SO_4_ to each well. Absorbance was measured with the help of ELISA plate reader at 450 and 620 nm.

### 4.11. Inhibition of Endothelial Cell Proliferation by VEGFR1(D1-D3)-Fc

Angiogenic factors including VEGF can promote cell proliferation in endothelial cells. Here, the inhibitory effect of the anti-angiogenic molecule, VEGFR1(D1–D3)-Fc, in inhibiting VEGF_165_-induced proliferation of human umbilical vein endothelial cells (HUVEC) was studied to confirm its biological activity. HUVEC (5000 cells per well) were seeded in wells of a 96-well microtitre plate in 100 µL assay medium containing 2% serum. Neutralization mixes containing VEGF_165_ (25 ng/mL) alone and in combination with different concentrations of VEGFR1(D1–D3)-Fc (5, 10, 50, 100, 150, 200, 250 ng/mL) were incubated at 37 °C in a 5% CO_2_ incubator for 3 h before being added to experimental wells containing cells. The plate was incubated at 37 °C for 72 h after which cell proliferation was quantitated by using the alamar blue method of detection. The fluorescence (relative fluorescence units, RFU) was measured at an excitation wavelength of 544 nm and an emission wavelength of 590 nm using a BMG Lab tech Polar star OPTIMA plate reader.

### 4.12. Inhibition of Endothelial Tube Formation by VEGFR1(D1-D3)-Fc

The ability of endothelial cells to form a three-dimensional capillary/tube-like network is a specialized function of these cells [27]. Inhibition of angiogenesis can be assessed *in vitro* by the inability of an angiogenesis inhibitor, such as VEGFR1(D1–D3)-Fc, to form such tubes in endothelial cell lines. Matrigel (50 µL) was added to wells of a 96-well plate and then incubated at 37 °C for 1 h to allow optimal well coating. Human umbilical vein endothelial cells (HUVEC), previously starved overnight, were seeded at a density of 10,000 cells in 50 µL per well in matrigel-coated wells in medium containing pre-incubated mix of VEGF_165_ at 50 ng/mL concentration and various concentrations of the VEGFR1(D1–D3)-Fc protein (5, 25, 100, 250 µg/mL). Plates were incubated at 37 °C for 4–6 h in a humidified 5% CO_2_ incubator after which, wells were viewed for tube formation at various time-points with an inverted microscope under bright field illumination and images acquired and processed with Image J software. An angiogenic parameter, namely total capillary length, per field, was evaluated. Results scored from two random fields in duplicate wells were averaged.

## 5. Conclusions

Fc-fusion proteins have a bright future in biotherapeutic industry owing to their vast repertoire of therapeutic targets, improved half-life and serum stability over monoclonal antibodies, application to the broad range of diseases, considerable success in the number of products so far approved and global sale values. Our recombinant Fc-fusion protein of interest, VEGFR1(D1–D3)-Fc, exhibits clipping in mammalian cell culture. In the current study, several parameters were evaluated including the addition of protease inhibitors in culture media, lowering of culture temperature and duration of culture, in a bid to reduce proteolysis of the fusion protein. It was observed that proteolysis could be reduced to a large extent by growing the cells in CDCHO media containing ferric citrate or protease inhibitor cocktail, as well as lowering of culture incubation temperature from 37 to 30 °C. The purified protein containing 5% degraded low molecular weight species, was overall stable with properly folded secondary and tertiary structures and had a strong affinity towards its biological ligands. *In vitro* bioassays using human endothelial cells indicated that the protein was biologically active. Further, *in vivo* studies need to be carried out in mice xenograft tumor models in order to assess the functional anti-tumor and anti-angiogenic activities of this fusion protein. Hence, it can be concluded from these studies that the protein, by virtue of its strong VEGF binding and inhibitory abilities, can be a promising therapeutic approach in diseases where VEGF-induced angiogenesis and abnormal endothelial proliferation play a key role.

## Supplementary Materials

Supplementary materials can be found at http://www.mdpi.com/1422-0067/17/6/913/s1.
