# Supplementary material for: Studies to Prevent Degradation of Recombinant Fc-Fusion Protein Expressed in Mammalian Cell Line and Protein Characterization
Source: Int J Mol Sci. 2016 Jun 9;17(6):913. doi: 10.3390/ijms17060913 (PMC4926446; doi:10.3390/ijms17060913)
Supplement: Supplementary file 1 [file ijms-17-00913-s001.pdf]

Supplementary Material: Studies to Prevent Degradation of Recombinant Fc-Fusion Protein Expressed in Mammalian Cell Line and Protein Characterization

Sanjukta Chakrabarti, Colin J. Barrow, Rupinder K. Kanwar, Venkata Ramana and Jagat R. Kanwar

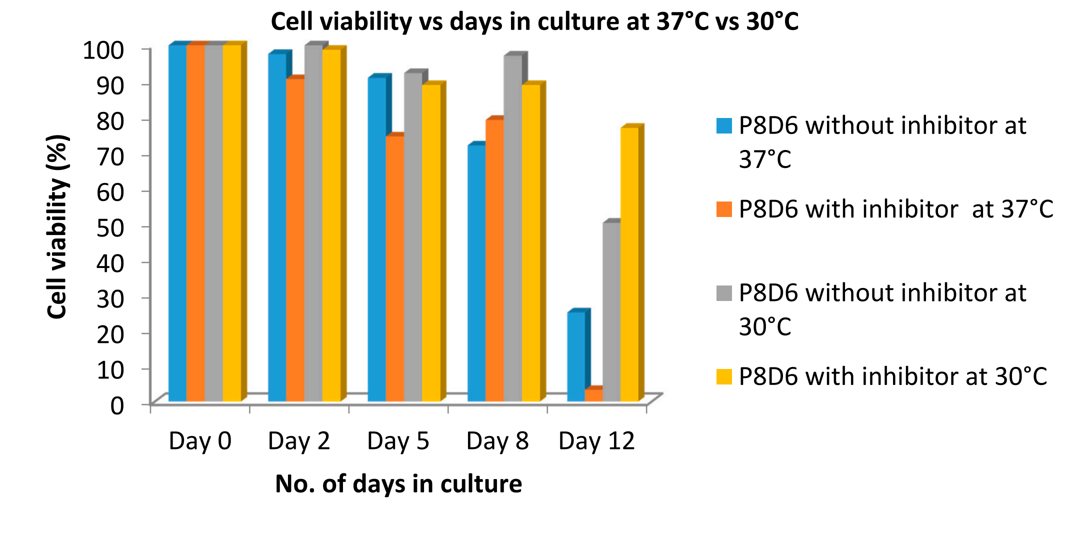

Figure S1. Graphical representation of cell viability vs. number of days in culture for VEGFR1 (D1–D3)-Fc clone in at 37 and 30 °C in presence and absence of protease inhibitor cocktail. The cells for each experimental condition, were grown in T-175 flasks in 50 mL CDCHO medium.

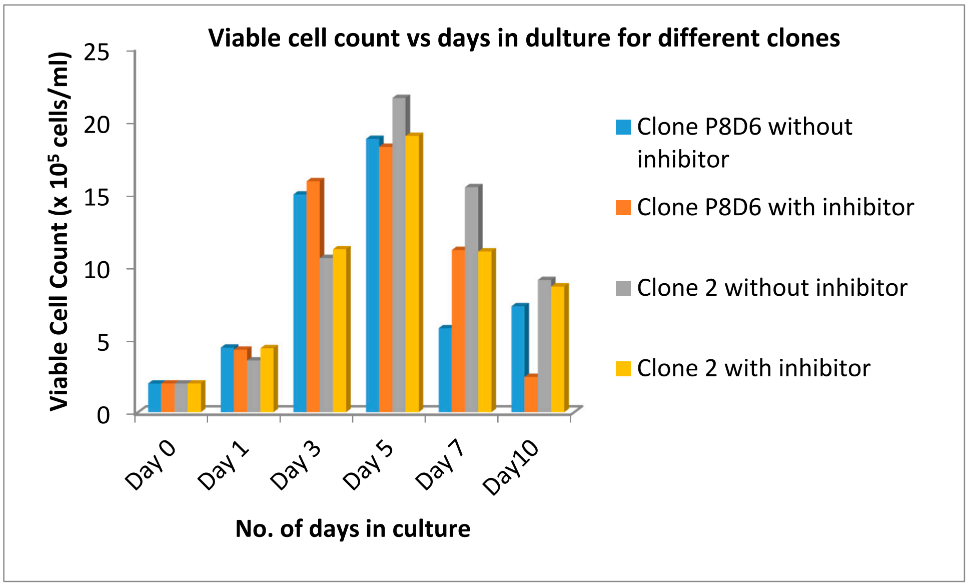

Figure S2. Graphical representation of viable cell count vs number of days in culture for two different clones. The cells for each experimental condition, were grown in T-25 flasks in 10 mL CDCHO medium.

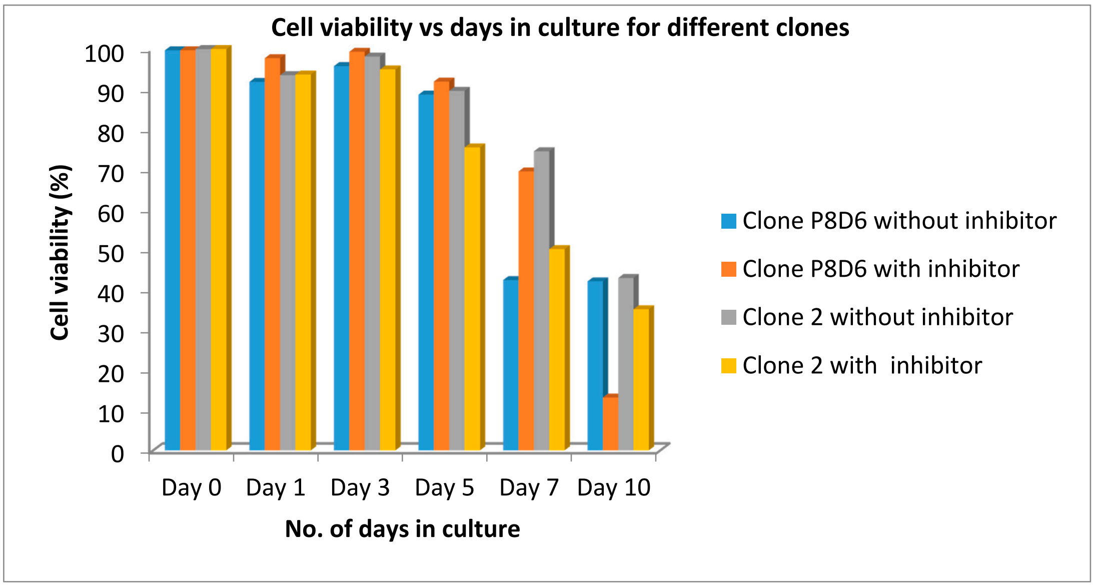

Figure S3. Graphical representation of cell viability vs. number of days in culture for two different clones. The cells for each experimental condition, were grown in T-25 flasks in 10 mL CDCHO medium.

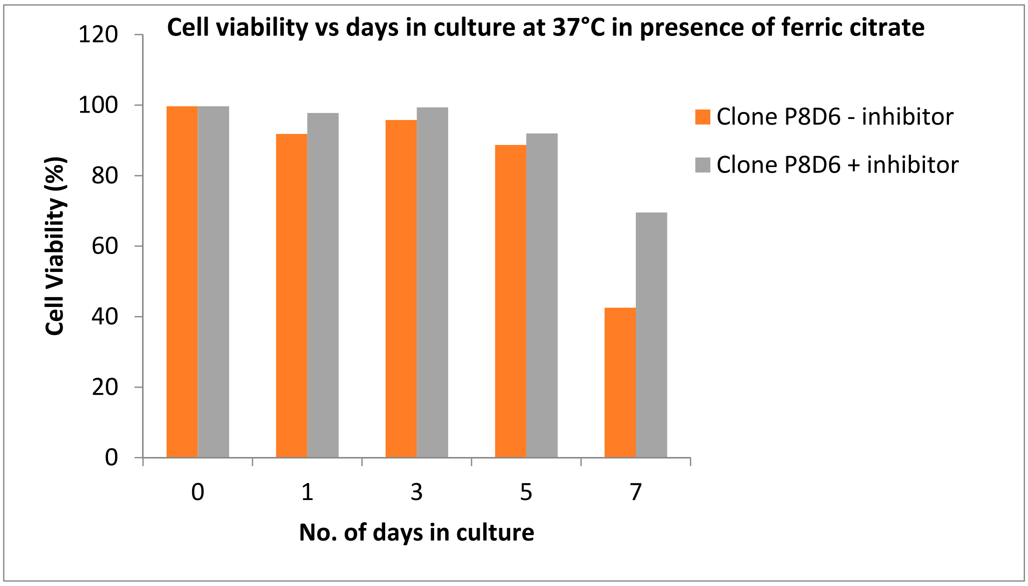

Figure S4. Graphical representation of cell viability *vs.* number of days in culture for VEGFR1 (D1–D3)-Fc clone P8D6 at 37 °C in presence and absence of ferric citrate (10×). The cells for each experimental condition, were grown in T-175 flasks in 50 mL CDCHO medium.
